# Impairment of Pneumococcal Antigen Specific Isotype-Switched Igg Memory B-Cell Immunity in HIV Infected Malawian Adults

**DOI:** 10.1371/journal.pone.0078592

**Published:** 2013-11-04

**Authors:** Oluwadamilola H. Iwajomo, Adam Finn, Abiodun D. Ogunniyi, Neil A. Williams, Robert S. Heyderman

**Affiliations:** 1 School of Cellular and Molecular Medicine, University of Bristol, Bristol, United Kingdom; 2 Malawi Liverpool Wellcome Trust Clinical Research Programme, University of Malawi College of Medicine, Blantyre, Malawi; 3 Research Centre for Infectious Diseases, School of Molecular and Biomedical Science, The University of Adelaide, Adelaide, Australia; University of Cape Town, South Africa

## Abstract

Pneumococcal disease is associated with a particularly high morbidity and mortality amongst adults in HIV endemic countries. Our previous findings implicating a B-cell defect in HIV-infected children from the same population led us to comprehensively characterize B-cell subsets in minimally symptomatic HIV-infected Malawian adults and investigate the isotype-switched IgG memory B-cell immune response to the pneumococcus. We show that similar to vertically acquired HIV-infected Malawian children, horizontally acquired HIV infection in these adults is associated with IgM memory B-cell (CD19^+^ CD27^+^ IgM^+^ IgD^+^) depletion, B-cell activation and impairment of specific IgG B-cell memory to a range of pneumococcal proteins. Our data suggest that HIV infection affects both T-cell independent and T-cell dependent B-cell maturation, potentially leading to impairment of humoral responses to extracellular pathogens such as the pneumococcus, and thus leaving this population susceptible to invasive disease.

## Introduction

The burden of invasive pneumococcal disease (IPD) is high in many African countries, including Malawi, where a high prevalence of HIV infection is associated with increased rates and severity of IPD in both adults and children [1], [2], [3]. Pneumonia, pneumococcal bloodstream infection and meningitis amongst HIV-infected individuals are all common reasons for adult hospital admissions in this setting [1], [4]. In Malawian children, we have previously demonstrated a shift in the B-cell compartment toward an apoptosis-prone phenotype (CD19^+^ CD10^−^ CD21^lo^) evident early in HIV disease progression, associated with reduced numbers of pneumococcal protein antigen–specific memory B-cells [5] which may in part explain their susceptibility to IPD.

We have recently shown that even in asymptomatic HIV-infected Malawian adults, pneumococcal-specific interferon–gamma (IFN-γ)-mediated CD4 T-cell effector memory and CD4 T-cell central memory proliferative responses are impaired [6]. An immune defect that does not fully resolve following antiretroviral therapy (ART) [7]. In other settings, HIV-infected adults with chronic infection or advanced disease have been shown to have an over representation of apoptosis prone immature transitional B-cells (CD10^+^ CD27^−^) and mature activated B-cells (CD10^−^ CD21^lo^) [8], [9], [10]. The mature activated B-cells have increased expression of CD95 and are notably susceptible to CD95 ligand-mediated apoptosis, while the immature transitional B-cells are notably susceptible to intrinsic apoptosis, expressing very low levels of anti-apoptotic Bcl-2 proteins [8], [9], [10], [11]. It is uncertain, however, whether these B-cell defects occur in African adults or whether they impact on antigen-specific immunity. We have therefore investigated whether in comparison to children who have acquired HIV perinatally (a critical time for the development of the B-cell compartment), Malawian adults are equally susceptible to HIV-mediated B-cell dysfunction. We comprehensively characterized the B-cell subset profile of HIV-infected Malawian adults and then investigated whether the dysregulation identified was associated with loss of isotype-switched IgG memory B-cells to pneumococcal protein antigens.

## Materials and Methods

### Study Participants

Otherwise healthy HIV-infected adults aged between 18 and 55 years old, with clinical features of WHO stage I were recruited from the voluntary counselling & testing (VCT) and ART out-patients clinics of Queen Elizabeth Central Hospital, Malawi following informed consent [12]. The volume of blood collected from these adults ranged from 30 – 50 mL. All participants were ART treatment naïve. Healthy controls with no clinical features of HIV were recruited by advertisement in the hospital. All controls were counselled and written consent was sought for HIV testing. HIV seropositivity was confirmed using two complementary HIV rapid antibody tests Unigold™ (Trinity Biotech, Ireland) and Determine™ (Abbott, Japan) according to manufacturers’ instructions. In the case of disparate results, a third test was performed using Bioline test kit (Standard Diagnostics Inc, Korea). Pregnant women were excluded from the study. None of the participants received a pneumococcal vaccine. Nasopharyngeal swabs were collected from study participants for the identification of *Streptococcus pneumoniae* [13]. In order to exclude malaria as a confounding factor in the immunological assays, all study participants were tested for malaria. This study complies with relevant guidelines and institutional practices of the Malawi-Liverpool Wellcome Trust Clinical Research Programme and the University of Malawi College of Medicine and was approved by the College of Medicine Research Ethics Committee (P.03/08/626).

### Immunophenotyping

The proportions and absolute numbers of B- and T-cells were estimated from EDTA whole blood samples by flow cytometry as previously described [5]. Briefly, fluorescein isothiocyanate (FITC)-labeled anti-CD19, and anti-CD21; phycoerythrin (PE)-labeled anti-CD8, anti-CD27, and anti-IgD; peridinin chlorophyll protein (PerCP)-labeled anti-CD3 and anti-CD19; and allophycocyanin (APC)-labeled anti-CD4, anti-CD10, and anti-CD27 were used for comprehensive phenotyping in the flow cytometric assays. A minimum of 100,000 events was acquired within the lymphocyte gate using CellQuest Pro software on a 4-color flow cytometer (BD FACSCalibur). Results were analysed using FlowJo software, version 7.2.2 (Tree Star).

### PBMC Enzyme-Linked Immunospot Assay

As previously described [5], memory B-cell numbers were enumerated using an enzyme-linked immunospot (ELISPOT) assay, following stimulation of freshly isolated peripheral blood mononuclear cells (PBMCs) in culture for 6 days in the presence of a combination of 1/100,000 standardized pansorbin cells (heat-killed, formalin-fixed *Staphylococcus aureus,* Cowan 1 strain), 1 μg/mL phosphothiolated CpG oligoneucleotide 2006 and 1/1000 pokeweed mitogen extract. This is an adaptation of the ELISPOT method of Crotty and is a sensitive technique able to detect antigen and isotype specific memory B-cells in peripheral blood that has differentiated into antibody secreting cells *in vitro* [14]. Immune memory to Choline binding protein A – CbpA, Pneumococcal surface protein A – PspA, Pneumolysin – Ply and Pneumococcal surface antigen A – PsaA [15], [16], [17], [18] was then enumerated using the AID ELISPOT reader and analysis software version 4.0 (AID).

### Statistical Analysis

We performed statistical analysis using Stata (version 10) and GraphPad Prism (version 4.0) software. Wilcoxon-Mann-Whitney *U*-test was used to perform the comparison between HIV^−^ and HIV^+^ adults. The Fisher’s exact test of probability was used for categorical data. Data were expressed as median values and interquartile ranges (IQR), and differences after comparisons were considered statistically significant if *P*<0.05. The association between two parameters was determined by Spearman’s correlation using Stata.

## Results

### Baseline characteristics of the study participants

Forty three HIV^+^ adults (median age 37, IQR 27 – 42 years) were recruited into the study and stratified into 2 groups comprising 17 participants who had CD4^+^ counts >350cells/µl (Group I) and 26 with CD4^+^ counts ≤350cells/µl (Group II), as ≤350cells/µl was the accepted threshold for initiation of ART in adults recommended by the WHO at the time of the study [12]. 44 HIV^−^ healthy control adults (median age 31, IQR 25 – 40 years) were also recruited. Demographic characteristics of study participants are shown in Table 1. Both CD19^+^ B-cell and CD4^+^ T-cell counts & percentages were lower in HIV^+^ adults compared to HIV^−^ controls. Also as expected, median CD8^+^ T-cell counts and percentages were higher in the HIV^+^ group than the controls. As we have previously observed in asymptomatic individuals [6], there was no statistically significant difference observed between pneumococcal carriage rates in HIV^+^ (7%) and HIV^−^ adults (12%) (Table 1).

**Table 1:** Baseline demographic and immune parameters of HIV-infected and uninfected Malawian adult study participants.

	HIV^−^ (n = 44)	All HIV^+^ (n = 43)	Significance of difference between HIV^−^ and all HIV^+^ *P values*	HIV^+^ Group I CD4>350 (cell/µl) (n = 17)	HIV^+^ Group II CD4≤350 (cell/µl) (n = 26)
Age, years	31 (25 – 40)	37 (27 – 42)	P = 0.1 (ns)	39 (30 – 41)	36 (26 – 44)
Female (%)	19/44 (43)	25/43 (58)	P = 0.2 (ns)	10/17 (59)	15/26 (58)
% CD4^+^ T cells	41.7 (34.7 – 45.9)	16.6 (9.1 – 24.5)	P<0.0001	24.5 (18.7 – 28.0)	11.9 (7.3 – 16.6)
CD4^+^ T cell count (cell/µl)	795 (662 – 927)	272 (154 – 442)	P<0.0001	459 (403 – 648)	173 (95 – 272)
% CD8^+^ T cells	28.4 (23.8 – 32.3)	55.1 (45.5 – 61.2)	P<0.0001	47.7 (38.5 – 55.9)	59.3 (47.3 – 65.7)
CD8^+^ T cell count (cell/µl)	536 (427 – 687)	843 (547 – 1307)	P = 0.0001	1041 (636 – 1234)	832 (526 – 1315)
% CD19^+^ B cells	9.8 (7.5 – 11.1)	7.6 (5.5 – 10.1)	P = 0.01	6.8 (5.8 – 10.2)	7.9 (5.2 – 10.2)
CD19^+^ B cell count (cell/µl)	189 (147 – 235)	116 (88 – 177)	P = 0.001	134 (112 – 308)	105 (83 – 168)
Malaria (%)	1/44 (2)	0/43 (0)	P = 0.9 (ns)	0/17 (0)	0/26 (0)
† *S. pneumoniae* carriage (%)	5/43 (12)	3/41 (7)	P = 0.7 (ns)	0/17 (0)	3/24 (13)

Median and interquartile ranges (IQR) for age and immunological data are shown. Statistical significance of differences between the HIV negative group and all HIV-infected adults was tested using Fisher’s exact test (for categorical variables) or Mann Whitney *U* test (for continuous variables). † Data on *S. pneumoniae* carriage are missing for some patients for technical reasons.

### B-cell immunophenotype in HIV-infected adults

**Figure 1:**
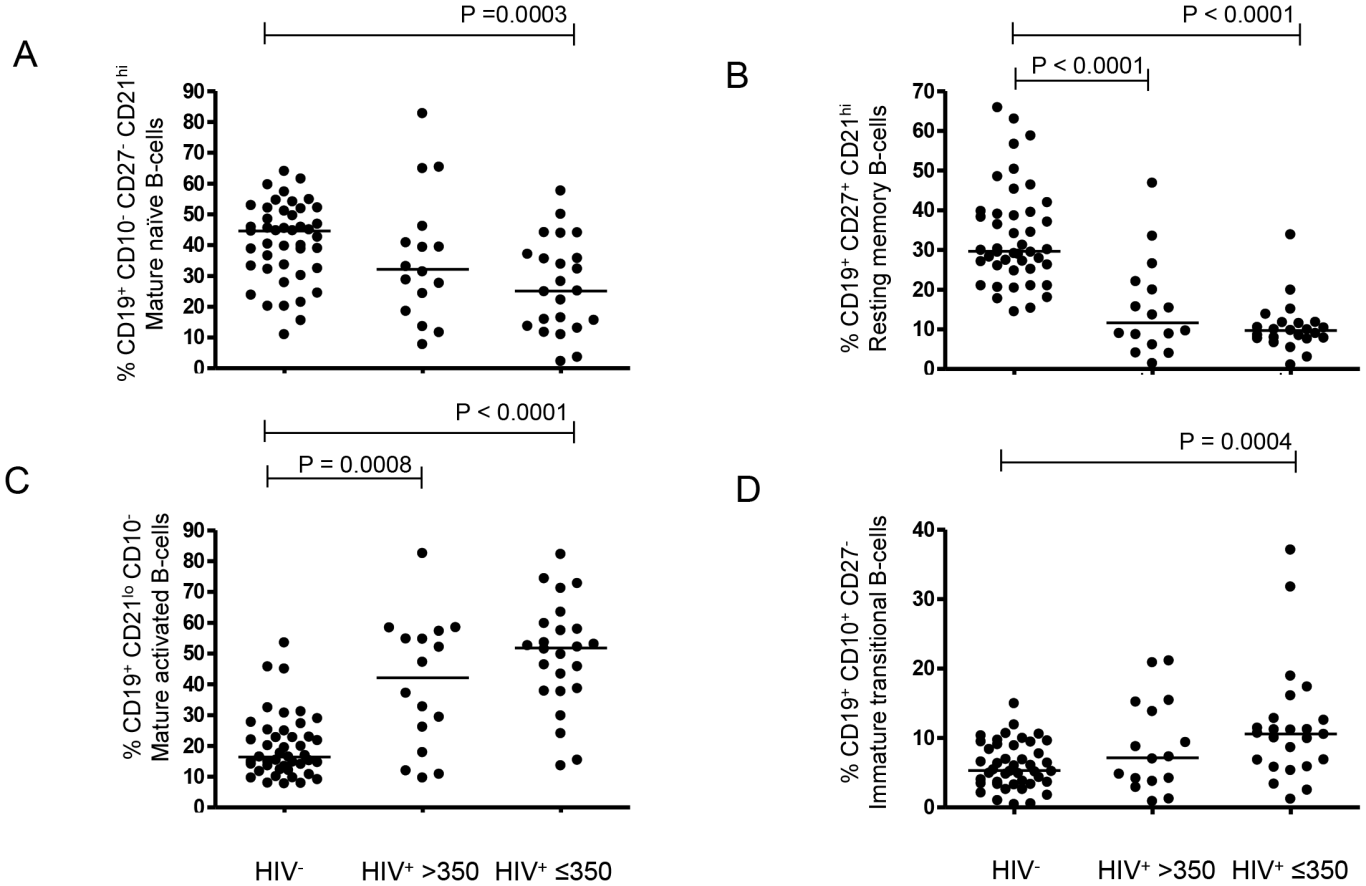
Loss of resting-memory B-cells and overrepresentation of apoptosis-prone mature-activated B-cells occurs early in HIV infection. Percentages of circulating (A) mature naïve (CD19^+^ CD10^−^ CD27^−^ CD21^hi^) (B) resting memory (CD19^+^ CD27^+^ CD21^hi^) (C) mature activated (CD19^+^ CD21^lo^ CD10^−^) and (D) immature transitional (CD19^+^ CD10^+^ CD27^−^) B-cells measured using flow cytometry in HIV^−^ (n = 44), HIV^+^ >350 (n = 16) and HIV^+^ ≤350 (n = 24) adults. Horizontal bars represent median values. Statistical significance of differences between groups was assessed using the Mann Whitney *U* test.

Consistent with our previous findings in children [5], both mature naïve (CD19^+^ CD10^−^ CD27^−^ CD21^hi^) and resting memory (CD19^+^ CD27^+^ CD21^hi^) B-cells were depleted in Malawian HIV^+^ adults, all of whom were in early stage disease by clinical criteria (WHO clinical stage I) compared to HIV^−^ controls (Figure 1A-B). In addition, the proportions of apoptosis prone mature activated (CD19^+^ CD21^lo^ CD10^−^) B-cells were significantly higher in the HIV^+^ cohort (Figure 1C). Apoptosis prone immature transitional (CD19^+^ CD10^+^ CD27^−^) B-cells were also significantly higher in HIV^+^ adults (Figure 1D). On stratifying HIV^+^ adults into two groups based on their CD4^+^ counts, depletion of resting memory B-cells was observed even in the group with relatively preserved CD4^+^ counts (Group I). An over-representation of mature activated B-cells was also evident in this group (Group I, Figure 1). Although a depletion of mature naïve B-cells and over-representation of immature transitional B-cells was observed in this group, it did not reach statistical significance (Group I, Figure 1A and D).

**Figure 2:**
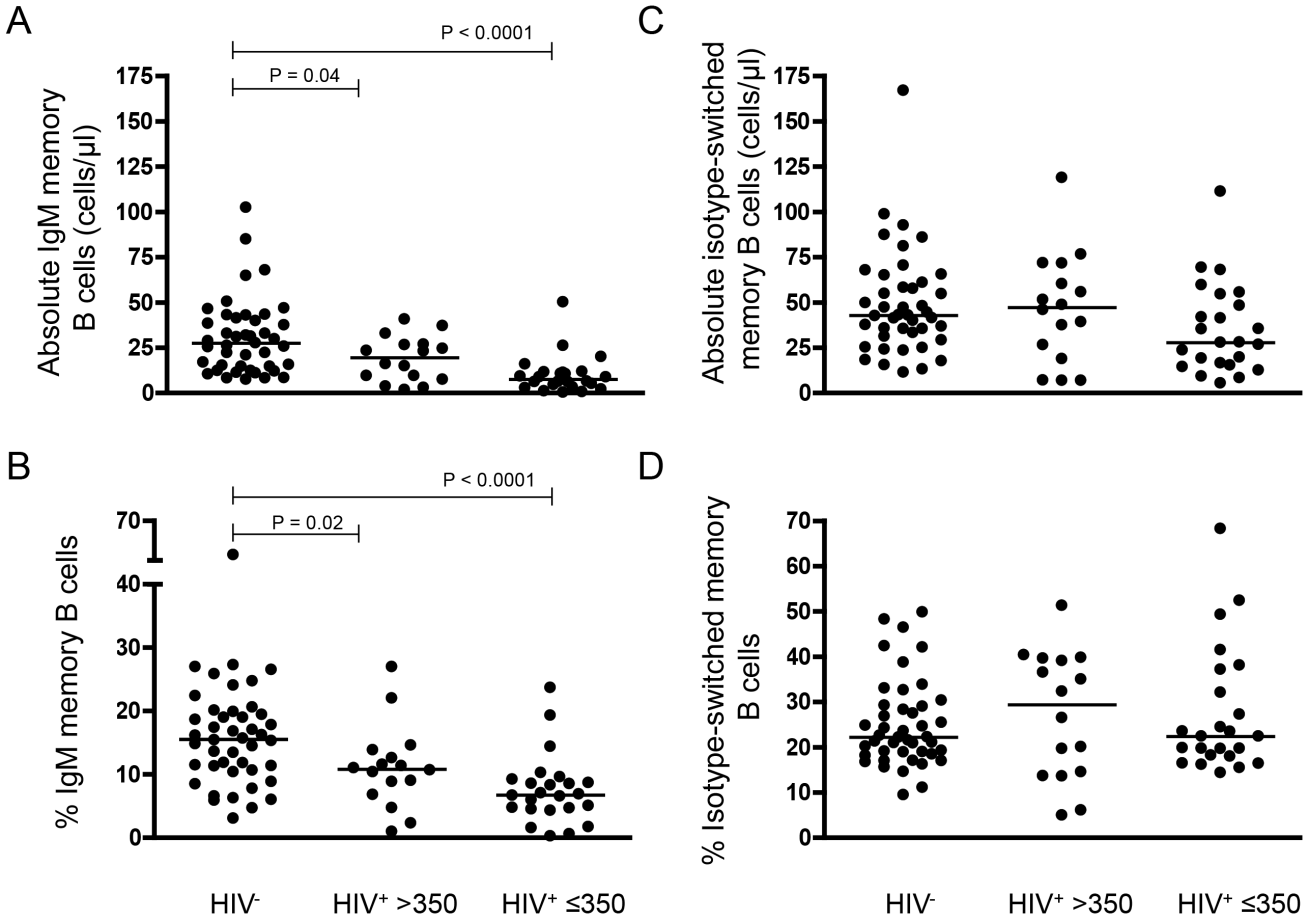
IgM memory B-cells are depleted while isotype-switched memory B-cells are maintained in HIV-infected Malawian adults. (A) IgM memory B-cells (CD19^+^ CD27^+^ IgD^+^) absolute numbers, (B) percentages and (C) isotype-switched memory B-cell (CD19^+^ CD27^+^ IgD^−^) numbers, (D) percentages in the circulation of HIV^−^ controls (n = 44), HIV^+^ >350 (n = 16) and HIV^+^ ≤350 (n = 24) adult. Horizontal bars represent medium values. Statistical significance of differences between groups was assessed using the Mann Whitney *U* test.

**Figure 3:**
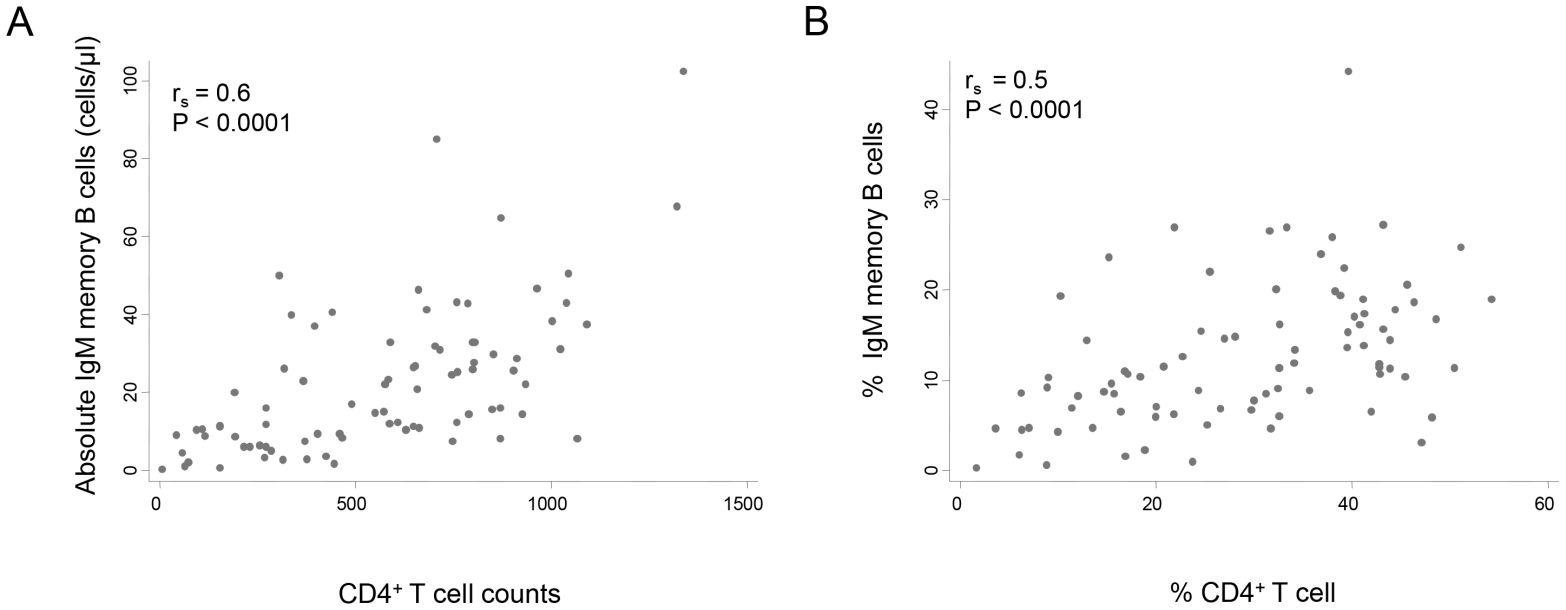
Correlation between circulating IgM memory B-cells and CD4^+^ T-cells. (**A**) Scatter plots showing absolute IgM memory B-cell numbers & CD4^+^ T-cell counts and (**B**) % IgM memory B-cells & CD4^+^ T-cells measured using flow cytometry. The association between the two parameters (IgM memory B-cells and CD4 counts) was determined by Spearman’s correlation using Stata (Version 10). Each point represents the result from one subject. r_s_ is the Spearman rank correlation coefficient.

The B-cell memory compartment was further investigated to delineate changes associated with HIV infection. The median IgM memory B-cell (CD19^+^ CD27^+^ IgD^+^) numbers and percentages in HIV^+^ adults were significantly lower than in HIV^−^ controls regardless of stratification (Figure 2A-B) and there was a moderate correlation between these IgM memory B-cell parameters and CD4^+^ T-cell numbers and percentages respectively (Figure 3). However, the median absolute numbers and percentages of peripheral blood isotype-switched memory B-cells (CD19^+^ CD27^+^ IgD^−^) in HIV^+^ adults was similar to that in HIV^−^ controls (Figure 2C-D).

### Pneumococcal protein antigen specific immunoglobulin G memory B-cells in the blood of HIV-infected adults

While IgM memory B-cells (directed largely against polysaccharide) are thought to contribute to the first-line defense against invasive disease caused by encapsulated bacteria, naturally acquired isotype-switched IgG memory B-cells to protein antigens are generally thought to be responsible for long-term serological memory. This isotype-switched memory is dependent on T-cell help, which we have previously shown to be defective in HIV-infected adults [6], [7].

**Figure 4:**
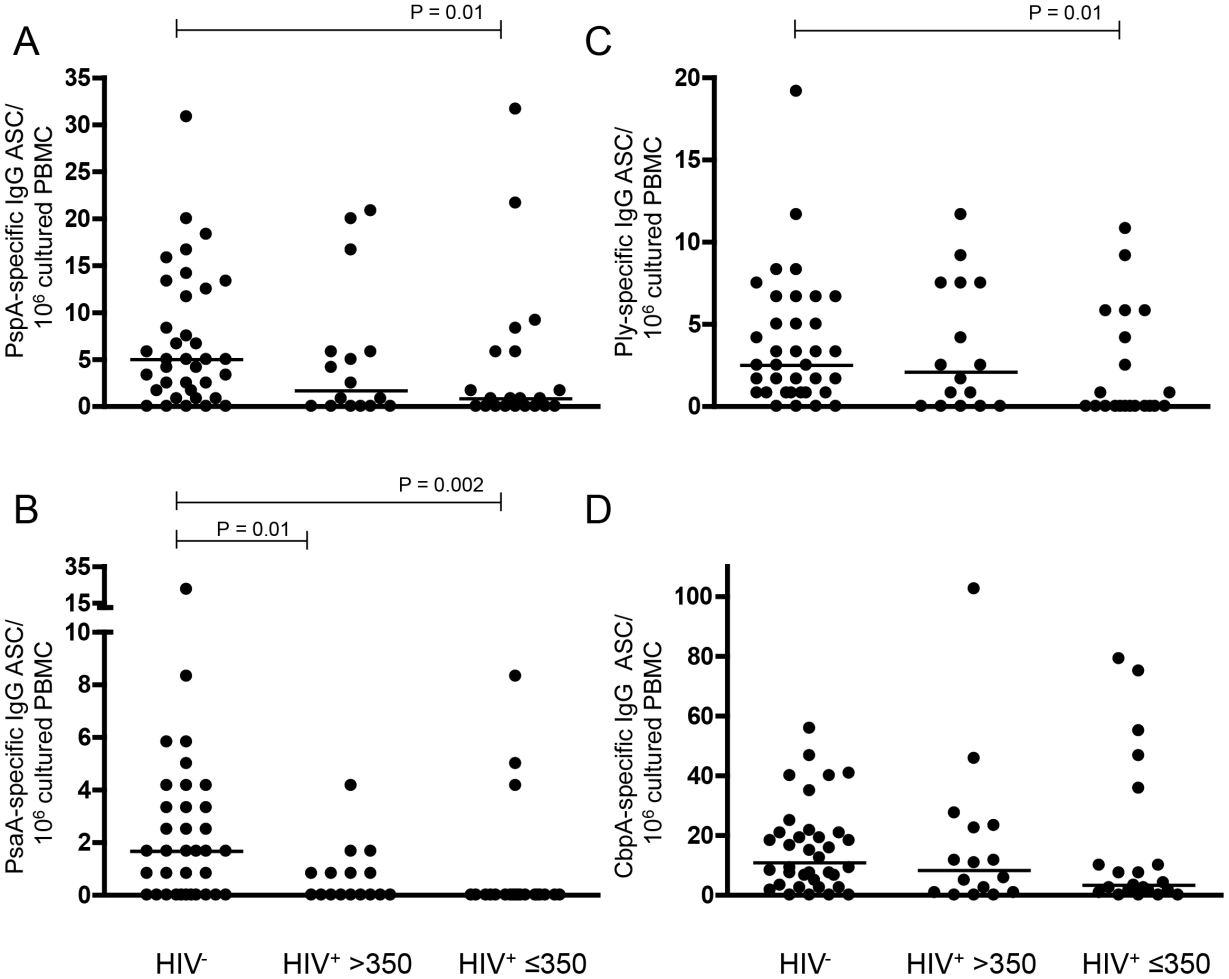
Loss of specific isotype-switched IgG memory B-cells in HIV-infected adults to pneumococcal protein antigens. Using an ELISPOT assay, following the expansion of memory B-cells with a cocktail of mitogens (SAC, PWM & CpG DNA) for six days, (**A**) Pneumococcal surface protein A (PspA), (**B**) Pneumococcal surface adhesion A (PsaA), (**C**) Pneumolysin (Ply), and (**D**) Choline binding protein A (CbpA) specific IgG antibody secreting cells (ASC) were enumerated in HIV^−^ controls (n = 36), HIV^+^ >350 (n = 16) and HIV^+^ ≤350 (n = 21) adults. Memory B-cell responses were expressed as numbers of ASC per million cultured PBMC seeded on the ELISPOT well. Each dot represents a mean of triplicates. Horizontal bars represent median values. Statistical significance of differences between groups was assessed using the Man Whitney *U* test.

We show that even though no decrease was observed in isotype-switched IgG memory B-cell proportions and numbers in Malawian HIV-infected adults, there was a functional impairment of these cells. More than twice the number of antigen-specific memory B-cells were found in the blood of HIV^−^ adults for all four antigens (pneumococcal surface protein A (PspA), pneumococcal surface adhesion A (PsaA), pneumolysin (Ply) and choline binding protein A (CbpA)), than in HIV^+^ adults. These results reached statistical significance for PspA and PsaA (P = 0.02, P = 0.0002 respectively; Figure 4). On stratifying HIV^+^ adults into two groups based on their CD4^+^ counts, depletion of PspA and PsaA-specific memory B-cells was observed even in the group with relatively preserved CD4^+^ counts (Group I), results reaching statistical significance for only PsaA. However, the differences were more extensive in the HIV^+^ group with CD4^+^ counts below 350 (Group II, Figure 4).

## Discussion

Progressive dysfunction of the humoral immune response is one of the hallmarks of HIV infection. Defects in B-cell immunity have been identified in HIV-infected adults with chronic infection or advanced disease in which apoptosis-prone immature transitional B-cells and mature activated B-cells are overrepresented [8], [10]. In line with these and our previous studies of HIV-infected Malawian children [5], we have found loss of total, naïve and resting memory B-cells together with an overrepresentation of apoptosis-prone B-cell subsets (immature transitional and mature activated B-cells), even among HIV-infected Malawian adults with relatively preserved CD4^+^ T-cell counts. These changes were associated with IgM memory B-cell depletion but not isotype-switched IgG memory B-cells, and impairment of specific B-cell memory to a range of pneumococcal proteins. Thus, taken together these data suggest that in minimally symptomatic HIV-infected individuals, the B-cell compartment is profoundly disrupted with a considerable shift in the distribution of normal B-cell subsets in the blood.

Although serotype-specific anticapsular antibodies are protective when induced by vaccination, naturally acquired antibodies against subcapsular pneumococcal proteins such as PspA, PsaA, Ply and CbpA, and interferon-γ and IL-17-producing CD4 T-cells are thought to be more important in natural immunity [19], [20], [21]. Impairment of B-cell memory to these key pneumococcal proteins observed in our HIV infected cohort, implicates defects in this B-cell immunity in IPD causation. We speculate that the impact of HIV on the immune memory responses to PspA and PsaA versus CbpA and Ply could amongst other things be due to, immunogenicity, differential T-cell defects and antigen expression during colonisation. It appears that at this stage of HIV-infection, pneumococcal colonisation is controlled, however, the ability to control nasopharyngeal carriage of the pneumococcus deteriorates as HIV disease progresses and does not reverse with ART therapy [7]. This highlights the need to study the functionality and duration of immune memory to novel candidate pneumococcal protein vaccine antigens in immunocompromised populations in order to optimize their effectiveness.

The origin and functionality of CD19^+^ CD27^+^ IgM^+^ IgD^+^ memory B-cells remain contentious. Based on their expression of the memory B-cell marker CD27 and the presence of *IGV* genes exhibiting few somatic mutations, (classically restricted to postgerminal center memory B-cells), these lymphocytes were initially thought to be non-switched memory IgM^+^ IgD^+^ B-cells [22]. Alternatively, it is now more widely believed that this IgM memory B-cell subset is germinal center-independent, emerging from the splenic marginal zone with a prediversified immunoglobulin repertoire. Following Toll-like receptor (TLR)-engagement, they are involved in the thymus-independent production of natural antibodies, the first line defence against encapsulated bacteria [22], [23], [24]. Indeed some studies have shown a strong correlation between the loss of IgM memory B-cells and reduced antibody levels to pneumococcal polysaccharides [24], [25]. In studies of polysaccharide vaccine antigens [26], we have found that antigen-specific IgM memory B-cells are not readily distinguishable from a large non-specific antibody response using the same polyclonal stimulated ELISPOT approach, which generates IgM antibodies to both vaccine and non-vaccine antigens regardless of vaccination status (unpublished data).

The exact mechanisms involved in the B-cell immunodeficiency observed in our minimally symptomatic HIV-infected adults are uncertain. In healthy individuals, following exposure to a T-cell dependent antigen, naïve B-cells can differentiate either into short-lived immunoglobulin secreting cells, long-lived plasma cells or memory B-cells [27], [28]. Our data do not exclude a functional T-cell defect or its effect on B-cell immunity. We have previously shown that asymptomatic HIV-infected adults show considerable T-cell immune dysregulation with impaired upregulation of the co-receptor CD154 [6]. We have also demonstrated that despite immune reconstitution with ART, CD4-mediated immunity is not fully restored [7]. In the present manuscript we show that even though no decrease was observed in isotype-switched IgG memory B-cell proportions and numbers in Malawian HIV-infected adults, there was a functional impairment of these cells. Whether the functional impairment results from the direct effects of HIV on the B-cell pool (e.g. from the transfer of negative factor from HIV-infected macrophages to B-cells [29]) or the indirect effects of impaired T follicular helper cells [30] remains to be determined. A possible confounding factor in our experiments may be lack of CD4 T-cell help in our PBMC cultures, amongst other things.

In conclusion, these data suggest that early in the course of disease progression, horizontally acquired HIV infection in adults primarily affects B-cell maturation after leaving the bone marrow, potentially leading to impairment of humoral responses to extracellular pathogens such as the pneumococcus and susceptibility to invasive disease. These defects appear to affect both T-cell independent and T-cell dependent B-cell-mediated immune defence against invasive pneumococcal disease. ART reduces polyclonal B-cell activation and normalizes the skewing of B-cell subsets [31]. How the early institution of ART in such individuals’ impacts on this B-cell immune defect requires further investigation.
